# Functional Analysis of Anti-cytokine Autoantibodies Using Flow Cytometry

**DOI:** 10.3389/fimmu.2019.01517

**Published:** 2019-07-12

**Authors:** Patricia A. Merkel, Terri Lebo, Vijaya Knight

**Affiliations:** ^1^Section of Allergy and Immunology, Department of Pediatrics, University of Colorado School of Medicine, Denver, CO, United States; ^2^Advanced Diagnostic Laboratories, National Jewish Health, Denver, CO, United States

**Keywords:** autoantibodies, cytokines, interferon gamma, GM-CSF, non-tuberculous mycobacteria, flow cytometry, phosphorylation

## Abstract

Autoantibodies to cytokines are increasingly being detected in association with immunodeficient, autoimmune and immune dysregulated states. Presence of these autoantibodies in an otherwise healthy individual may result in a unique phenotype characterized by predisposition to infection with specific organisms. The ability to detect these autoantibodies is of importance as it may direct treatment toward a combination of anti-microbial agents and immunomodulatory therapies that decrease autoantibody levels, thereby releasing the immune system from autoantibody-mediated inhibition. Ligand binding assays such as ELISA or bead multiplex assays have been used to detect these antibodies. However, not all anti-cytokine autoantibodies have demonstrable function *in vitro* and therefore their clinical significance is unclear. Assays that evaluate the functionality of anti-cytokine autoantibodies can supplement such ligand binding assays and add valuable functional information that, when viewed in the context of the clinical phenotype, may guide the use of adjunctive immunomodulatory therapy. This mini review provides an overview of anti-cytokine autoantibodies identified to date and their clinical associations. It also describes the use of flow cytometry for the functional analysis of anti-IFNγ and anti-GM-CSF autoantibodies.

## Introduction

The association of anti-cytokine autoantibodies (AAbs) with primary or acquired immunodeficiency, immune dysregulation and autoimmunity has been increasingly documented in literature (1–3). In fact, immunodeficiency or immune dysregulation due to autoantibodies that target specific cytokines or cytokine pathways now form a unique category in the latest International Union of Immunological Societies (IUIS) PID expert committee (EC)'s classification of immunodeficiencies (4). This category, termed “phenocopies of primary immunodeficiencies (PIDs),” includes acquired immunodeficiency due to certain anti-cytokine AAbs, notably to interferon gamma (IFNγ), interleukin-6 (IL-6), interleukin-17 (IL-17), interleukin-22 (IL-22), and Granulocyte Macrophage Colony Stimulating Factor (GM-CSF) that result in phenotypes similar to those that occur due to pathogenic variants in genes encoding either these specific cytokines, their receptors or molecules mediating cytokine signal transduction.

Anti-cytokine AAbs can be found in circulation and may mediate diverse infectious and/or immunological manifestations depending on the cytokine that they specifically target. Anticytokine AAbs may decrease bioavailability of cytokines by inhibiting binding to their cognate receptors or by sequestering the cytokine in high molecular weight complexes that subsequently undergo Fc-dependent degradation (5). Alternatively, these AAbs may prolong the action of the cytokine by forming immune complexes that promote increased cytokine activity through interaction with stimulatory receptors, FcγRII and FcγRIII. For example, IL8:IL8 AAb complexes have been shown to interact with FcγRIIa and increase IL-8 mediated neutrophil activity in acute respiratory distress syndrome (ARDS) (6).

The ability of anti-cytokine AAbs to mediate disease manifestations may depend on their ability to neutralize or potentiate cytokine function which in turn, may be dependent on the concentration of the AAb in circulation or in tissue, the avidity of the AAb, or the epitopes recognized by the AAb (5). In an analysis of anti-GM-CSF AAbs isolated from patients with Pulmonary Alveolar Proteinosis (PAP), Piccoli and colleagues showed that while monoclonal anti-Granulocyte Macrophage Colony Stimulating Factor (GM-CSF) AAbs showed diminished capacity to neutralize the cytokine in a bioassay, combinations of three non-competing anti-GM-CSF AAbs were effective in neutralizing the biological activity of GM-CSF. Additionally, healthy individuals were shown to have anti-GM-CSF AAbs at concentrations or in combinations that were non-neutralizing, whereas PAP patients generally had polyclonal antibodies at concentrations and titers that were capable of neutralizing the cytokine (5).

Anti-cytokine AAbs are generally polyclonal IgG in nature, however, low titer, non-neutralizing IgA AAbs to interleukin-10 (IL-10) have been detected in the serum of patients with inflammatory bowel disease (IBD) (7). Because the majority of IgA is secreted and only 15% of circulating immunoglobulins are of the IgA isotype, it is possible that these AAbs accumulate in inflamed tissue or at mucosal surfaces rather than in serum, suggesting that investigation of IgA AAbs should be perhaps be performed in relevant tissues or mucosal secretions.

Anti-cytokine AAbs can also be detected in a majority of healthy individuals and in therapeutic human immunoglobulin preparations (8). In fact, a study of over 8,000 healthy blood donors revealed that AAbs to IL-1α, IL-6, IL-10, and GM-CSF were not uncommon in healthy individuals and were noted to reach potentially neutralizing concentrations, chiefly in the case of anti-IL-6 AAbs (9). Although the significance of anti-cytokine AAbs in healthy individuals has not been definitively established, analysis of their functional activity, whether antagonistic to or agonistic with the biological activity of the cytokine is helpful to determine their significance in a disease setting (7, 10).

Under physiological conditions, anti-cytokine AAbs may potentially play a role in the regulation of biological activities of cytokines either by neutralizing excessive cytokine production or by prolonging the half-life of cytokines in circulation by forming cytokine-antibody immune complexes (6). This potential regulatory function is evidenced by the increase in levels of anti-cytokine AAbs with increasing amounts of cytokines (11). Under certain pathological conditions such as in rheumatoid arthritis (RA) or systemic lupus erythematosus (SLE), these AAbs have been observed to rise with decrease in clinical symptoms, suggesting that anti-cytokine AAbs may be used as tools to monitor severity or resolution of disease (12, 13).

There is growing evidence that suggests certain anti-cytokine AAbs play a direct pathogenic role in increasing susceptibility to infection or development of immune dysregulated states. For example, AAbs to interferon gamma (IFNγ), a cytokine responsible for protective immune responses against intracellular organisms, are associated with chronic, disseminated, treatment refractory infections with intracellular organisms such as mycobacteria (14) and AAbs to Granulocyte Macrophage Colony Stimulating Factor (GM-CSF), the principal orchestrator of maturation and function of pulmonary alveolar macrophages, are associated with autoimmune Pulmonary Alveolar Proteinosis (PAP) as well as with increased susceptibility to infection with Nocardia, Cryptococcus and Aspergillus species (5, 15).

In other clinical conditions, the presence of anti-cytokine AAbs might correlate with disease resolution; for example, an increase in anti-IL-1 AAbs correlates with milder disease course in rheumatoid arthritis (12), anti-interferon alpha (IFNα) AAbs have been noted to have an inverse correlation with disease severity in SLE (13), and high levels of anti-IFNγ AAbs correlate with resolution of Guillain-Barre Syndrome (16). The presence of the AAbs may also merely be an association rather than causative of disease (e.g., anti-Granulocyte Colony Stimulating Factor (G-CSF) AAbs in Felty's syndrome) (17).

The genetic and/or environmental factors that influence the development and potential pathogenicity of these anti-cytokine AAbs in an individual remain poorly defined. Understanding the true prevalence, titer and biological significance of anti-cytokine AAbs in healthy individuals or in specific disease cohorts is confounded by the diversity of techniques used to detect them. Therefore, it is important to measure not only the titer of the AAbs, but to also use functional assays, that may help to determine if the AAbs are functional and therefore more likely to be pathogenic. Appropriate recognition of these AAbs in the context of disease is also important because it may direct treatment toward a combination of adjunctive immunotherapy to modulate the AAb titer while continuing appropriate anti-microbial or other suitable therapy.

Table 1 lists anti-cytokine AAbs associated with various disease states identified to date and the assays, both binding and functional, that have been used to characterize these AAbs. Several of these AAbs may be associated with multiple disease states and may either lead to increased susceptibility to certain pathogens or may be immunoregulatory and dampen autoimmune-mediated disease symptoms. Therefore, the biological significance of anti-cytokine AAbs must be evaluated in the context of disease.

**Table 1:** Disease-associations of anti-cytokine AAbs and *in vitro* detection methods.

**Cytokine**	**Clinical Phenotype**	**Possible biological role**	**Assay**	**References**
**PRIMARILY INFECTIOUS MANIFESTATIONS**				
Interferon gamma (IFNγ)	Disseminated extra-pulmonary, NTM infections, infections with *Salmonella typhi*, Toxoplasma, CMV, reactivation of VZV. Likely immunomodulatory in Guillain-Barre Syndrome	Neutralizing, abrogates the IFNγ response, leading to compromised cellular immune responses. Neutralizes IFNγ thereby decreasing inflammation in GBS	Ligand-binding assay (ELISA) Functional, abrogation of p-STAT1 in human monocytes.	(1, 18, 19) (16)
Interleukin-17 (IL-17A, IL-17F)	APS-1, CMC	Neutralizing, abrogates IL-17 responses essential for anti-fungal immunity	ELISA Inhibition of IL-6 production by IL-17 responsive fibroblasts	(20, 21)
Interleukin-22 (IL-22)	APS-1, CMC	Found in association with anti-IL17 AAbs and may play a role in anti-fungal immunity. Not conclusively established.	Particle based ligand binding assay	(22, 23)
Granulocyte Macrophage Colony Stimulating Factor (GM-CSF)	Autoimmune PAP, intracellular infections with *Mycobacterium avium* and Cryptococcus, Nocardia, and Aspergillus species	Neutralizing, impaired alveolar macrophage development leading to compromised surfactant clearance, impaired macrophage, and neutrophil function	ELISA, proliferation of TF-1 cells in response to recombinant GM-CSF, Inhibition of p-STAT5 detection by flow cytometry	(5, 15, 24, 25)
Interleukin-12 (IL-12)	APS-1, thymoma associated autoimmune disease. Burkholdaria lymphadenitis (one documented case)	Neutralizing capability may increase susceptibility to intracellular organisms, however, biological role not conclusively established	Particle based ligand binding assay Inhibition of p-STAT4 in PHA-induced T cell blasts	(23, 26, 27)
**INFECTIOUS OR AUTOIMMUNE MANIFESTATIONS**				
Interleukin-6 (IL-6)	Documented association with systemic sclerosis, recurrent staphylococcal infections with low CRP levels.	Neutralizing, leads to decreased CRP levels, increased susceptibility to infection. May form stable complexes with IL-6 and contribute to disease progression in systemic sclerosis.	Luciferase immunoprecipitation (LIPS) ELISA, Western blot, inhibition of TF-1 cell growth Radioimmunoprecipitation	(28–30)
Interferon-alpha (IFN-α)	SLE, APS-1, Thymoma, immune deficiency associated with hypomorphic RAG mutations, NFKB2 mutations (one patient), IPEX syndrome	Neutralizing activity may increase susceptibility to infections. Neutralizing activity associated with reduction in disease severity in SLE, Sjogren's syndrome, and RA.	ELISA Viral growth inhibition Multiplex bead assay Inhibition of p-STAT1	(27, 31–33)
Granulocyte Colony Stimulating Factor (G-CSF)	Neutropenia, Felty's syndrome	May contribute to neutropenia through neutralization of G-CSF, however, robust evidence not available.	ELISA Western blotting Inhibition of proliferation of G-CSF receptor expressing 32D cell line.	(17)
Interleukin-1 (IL-1)	Pemphigus, psoriasis, rheumatoid arthritis, Sjogren's syndrome (non-destructive form of polyarthritis)	Shown to be neutralizing, negatively correlated with disease severity and may modulate disease.	Radioimmunoprecipitation, ELISA	(12, 34, 35)
B Cell Activating Factor (Baff)	Systemic Lupus Erythematosus, associated with CVID	Associated with decreased disease activity in SLE, but role unclear. Associated with CVID but does not correlate with pathogenesis of disease	ELISA	(36, 37)
**PRIMARILY AUTOIMMUNE OR IMMUNE DYSREGULATION**				
Tumor Necrosis Factor-alpha (TNF-α)	SLE, Multiple Sclerosis, psoriasis, RA	May play a role in disease modulation in SLE, RA, and Psoriasis. Role unclear in MS.	ELISA TNF-α induced apoptosis in U937 cells	(38)
Interleukin-8 (IL-8)	Acute Respiratory Distress Syndrome	Complexes with IL-8 thereby extending its proinflammatory activity including recruitment of neutrophils	ELISA to detect IL-8-anti-IL-8 complexes Ability to trigger neutrophil degranulation and release of superoxide	(10, 39)
Erythropoietin (EPO)	Acquired pure red cell aplasia (PRCA)	Neutralizing AAbs to exogenous recombinant EPO cross react with endogenous EPO, inhibiting growth of erythroid progenitor cells.	Radioimmunoprecipitation. Ability of serum from EPO-treated patients to inhibit the proliferation of erythroid progenitor cells from healthy donor bone marrow.	(40)
Osteopontin (OPN)	Rheumatoid arthritis, hepatocellular carcinoma, prostate cancer.	Unclear, may have a role in modulating disease activity in RA, Potential early serum biomarker for prostate cancer. Diagnostic and prognostic biomarker for hepatocellular carcinoma	ELISA Western blotting,	(41, 42) (41, 42)
Osteoprotegerin	Osteoporosis, celiac disease, increased bone resorption in rheumatoid arthritis	Biological role unclear.	Direct and Competitive ELISA	(43, 44)

*AAbs, Autoantibodies; NTM, Non-tuberculous mycobacteria; CMV, Cytomegalovirus; VZV, Varicella Zoster Virus; APS-1, Autoimmune Polyglandular Syndrome-1; CMC, Chronic Mucocutaneous Candidiasis; PAP, Pulmonary Alveolar Proteinosis; CRP, C-reactive protein; RAG, Recombination Activating Gene; NFKB2, Nuclear Factor Kappa B Subunit 2; IPEX, Immune dysregulation, polyendocrinopathy, enteropathy, X-linked; SLE, Systemic Lupus Erythematosus; RA, Rheumatoid Arthritis; CVID, Common Variable Immunodeficiency; PRCA, Pure Red Cell Aplasia; EPO, Erythropoietin*.

## Analysis of Anti-cytokine Autoantibodies

Although the association of anti-cytokine AAbs with immunodeficiency, dysregulation and autoimmunity is well-recognized, diagnostic testing for these AAbs is limited. Apart from validated diagnostic testing in a few clinical laboratories for the detection anti-GM-CSF AAbs in suspected or confirmed autoimmune PAP, anti-cytokine AAb testing is generally not routinely performed by most clinical laboratories. Analysis of anti-cytokine AAbs can be performed by laboratory-developed ELISA, radioimmunoassay, multiplex bead arrays or other ligand binding assays, and offers the ability to report an antibody titer and/or concentration. Ligand binding assays are, in general, high throughput, cost effective and automatable, and the methods can be fairly easily adapted by most clinical laboratories. Functional assessment of autoantibodies provides further information regarding their biological significance in the context of disease and requires demonstration of their neutralizing or potentiating capacity. These assays, while providing important functional information, are generally performed in laboratories that specialize in high complexity testing. Functional assessment of either the antagonistic or agonistic action of these AAbs has been performed in a variety of ways including demonstration of inhibition of proliferation of cytokine-responsive cell lines, inhibition of the ability of certain cytokines to restrict viral growth in permissive cell lines, inhibition of cytokine-driven differentiation of responsive cell lines, inhibition of cytokine-specific phosphorylation signals or potentiation of cellular function.

Flow cytometry is becoming more widely available in clinical laboratories and can be utilized to demonstrate inhibition of cytokine signaling pathways by neutralizing AAbs, and by inference, an indication of their ability to neutralize cytokine function *in vivo*. Although currently not widely available, flow cytometry based assays have been established for IFNγ and GM-CSF AAbs by exploiting knowledge of their signaling pathways. These assays and their relevance in diagnosis or monitoring disease states will be discussed below.

## Interferon Gamma AAbs

The key role of IFNγ in generation of protective immunity to mycobacterial infections and other intracellular infections is underscored by the fact that mutations of genes encoding *IFNGR1* and *IFNGR2*, (the ligand-binding and intracellular, signaling subunits, respectively, of the IFNγ receptor), and *STAT1* (Signal Transducer and Activator of Transcription 1), that is downstream of the IFNγ receptor, often lead to severe infections with intracellular organisms of low pathogenicity such as the *Bacille Calmette Guerin* (BCG) vaccine or non-tuberculous mycobacterial (NTM) species (45). Such infectious manifestations tend to occur in childhood. In adults, such infections are generally rare and if they occur, are generally associated with an acquired immune deficient state, such as HIV infection or immunosuppression following solid organ or hematopoietic stem cell (HSCT) transplant (46–50).

The initial identification of an acquired immune defect that disrupted the IFNγ pathway was published in 2004 and described an adult Filipino patient with high titer, neutralizing AAbs to IFNγ and associated extra-pulmonary NTM infection (51). Following this report, several cases of intracellular infections associated with AAbs to IFNγ have been documented in otherwise healthy individuals (14, 18, 52). The general clinical manifestation in these patients is extra-pulmonary, disseminated, treatment refractory infections with NTM, although *Salmonella typhi*, cytomegalovirus, cerebral toxoplasmosis and reactivation of varicella zoster virus (VZV) have been reported as well (53). The common features of this autoimmune phenomenon that contribute to an immune deficient state are that patients are, in general, otherwise healthy adults, predominantly Southeast Asian, female, and not obviously immunocompromised. IFNγ AAbs in these patients are neutralizing in nature and tend to be of a very high titer.

While the reason behind development of anti-IFNγ AAbs in certain individuals is not clear, recent data suggest that molecular mimicry and specific HLA types may play a role, therefore indicating that both environment and genetics may be responsible for this phenomenon. In 2013, Chih-Yu Chi et al. published studies showing a strong correlation between two HLA alleles, DRB1^*^16:02 and HLA-DQB1^*^05:02 and the occurrence of anti-IFNγ AAbs, suggesting a potential genetic basis for the development of these antibodies (54). Additionally, the group showed that the IFNγ epitope targeted by the anti-IFNγ AAbs was highly homologous to a stretch of amino acids in the Noc2 protein of Aspergillus spp (55). Together, these findings raise the possibility of molecular mimicry leading to development of a cross reactive antibody response to a self-antigen in the context of certain HLA types, leading to an acquired form of immunodeficiency.

## GM-CSF AAbs

PAP is a rare disease in humans and may be congenital, secondary or acquired (56). The acquired form of PAP has been shown to be due to AAbs to GM-CSF that neutralize the cytokine *in vivo*, compromising alveolar macrophage function and leading to accumulation of pulmonary surfactant in the lungs (5). Impairment of alveolar macrophage adhesion, chemotaxis, phagocytosis, and killing as well as neutrophil phagocytosis, adhesion, oxidative burst and bactericidal activity have been demonstrated in patients with acquired PAP, suggesting that neutralization of GM-CSF *in vivo* compromises critical functions in these cell types (57, 58). These observations also provide an explanation for the increased frequency of infections with opportunistic pathogens including *Mycobacterium avium* complex, Cryptococcus, Nocardia, Histoplasma and Aspergillus species in patients with PAP (59, 60). Anti-GM-CSF AAbs have also been described in otherwise immunocompetent patients with disseminated, extrapulmonary Nocardia infection and invasive aspergillosis, none of whom had an accompanying diagnosis of autoimmune PAP prior to presenting with these infections (15). In at least one of these patients, the presence of anti-GM-CSF AAbs predated clinical presentation, suggesting that these AAbs are likely to be causative of disease rather than a reaction to disease (15). It is currently unclear why certain individuals progress to PAP and others to increased susceptibility to infections with intracellular, opportunistic pathogens. These descriptions have added to the spectrum of clinical presentations in which the presence of anti-GM-CSF AAbs should be investigated (24).

## Flow Cytometry Analysis; Exploiting Cytokine Signaling Pathways

**Figure 1:**
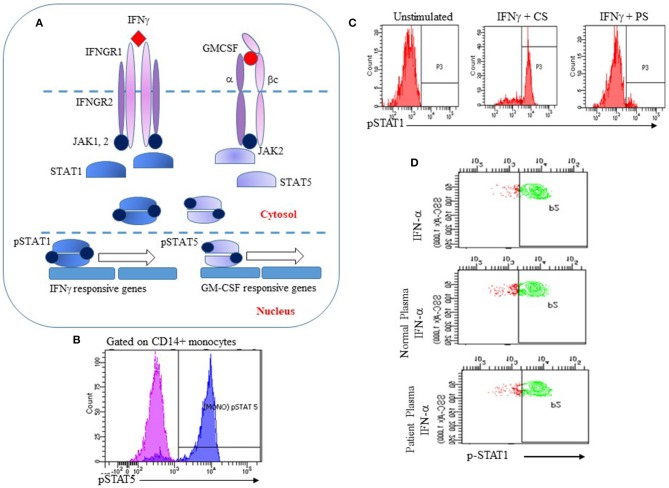
Utility of the IFNγ and GM-CSF signaling pathways for analysis of anti-IFNγ or anti-GM-CSF AAbs. **(A)** IFNγ or GM-CSF bind to their cognate receptors causing Jak1 or 2 to be phosphorylated, leading to phosphorylation and dimerization of STAT1 or STAT5, respectively. Phosphorylated STAT1 and STAT5 dimers translocate to the nucleus and initiate transcription of IFNγ or GM-CSF responsive genes respectively; **(B)** Recombinant GM-CSF induces phosphorylation of STAT5 in human monocytes (pink: unstimulated cells; blue: stimulated cells); **(C)** Human PBMCs were incubated with recombinant IFNγ and control serum (CS) or serum from a patient with disseminated NTM (PS). PS strongly inhibited IFNγ-induced phosphorylation of STAT1; **(D)** Specificity of the flow cytometry assay for detection of anti-IFNγ AAbs. Human PBMC were stimulated with IFNα alone, or with IFNα and control serum or patient serum. Patient serum did not inhibit IFNα-induced phosphorylation of STAT-1 indicating that p-STAT1 inhibition was specific to IFNγ AAbs.

Interferon gamma, a type II interferon secreted chiefly by T lymphocytes and Natural Killer cells, plays a critical role in host defense against intracellular pathogens such as mycobacteria and salmonella. The IFNγ receptor is highly expressed on antigen presenting cells (monocytes, macrophages and dendritic cells) and to a lesser extent on lymphocytes (61). Binding of IFNγ to its cognate receptor leads to activation of Janus kinases 1 and 2 (Jak 1 and 2) followed by phosphorylation of STAT1 on a single tyrosine residue (Y701). Phosphorylated STAT1 dimerizes and is translocated to the nucleus where it initiates transcription of IFNγ regulated genes (Figure 1A) (61).

GM-CSF mediates its functions through binding to its receptor, a heterodimer that is comprised of a specific a subunit (GMRα) and a dimeric b subunit (βc) that is shared with other cytokines of the βc family. Following binding of GM-CSF to its receptor, the βc subunit associates with Jak2 that then leads to phosphorylation of STAT5, leading to intranuclear translocation and transcription of GM-CSF regulated genes (Figure 1A) (62). Compromised GM-CSF signaling leads to functional deficits in multiple cell types including macrophages and neutrophils.

The IFNγ and GM-CSF signaling pathways have been used to develop flow cytometry based strategies for the functional detection of autoantibodies to these cytokines. The basis of this assay is a short stimulation of isolated peripheral blood mononuclear cells (PBMC) from a healthy donor with either recombinant human IFNγ or GM-CSF to induce phosphorylation of STAT1 or STAT5, respectively. Isolated PBMCs are first stained with fluorophore-conjugated anti-CD14 to identify monocytes. Stained PBMCs are then incubated at 37°C with an optimized concentration of either recombinant IFNγ or GM-CSF for 10–15 min. PBMCs are fixed immediately at the end of the stimulation period with 4% paraformaldehyde to preserve their phosphorylation status and permeabilized using methanol to allow staining for intracellular proteins. Intracellular phosphorylated STAT1 (p-STAT1) or STAT5 (p-STAT5) is detected in monocytes by staining PBMCs with fluorophore conjugated monoclonal antibodies to p-STAT1 (Tyr701) or p-STAT5 (Tyr694) (Figure 1B). Addition of serum or plasma suspected to contain AAbs to IFNγ or GM-CSF to this system is expected to result in decreased fluorescence for p-STAT1 (Figure 1C) or p-STAT5. Because IFN-α also utilizes p-STAT1 for signal transduction, PBMC stimulation with IFN-α is performed to demonstrate specificity of the assay for anti-IFNγ AAbs (Figure 1D).

## Flow Cytometry Analysis: Limitation and Challenges

As with most functional assays, flow cytometry analysis of p-STAT1 and p-STAT4 may be affected by factors other than specific AAbs in patient serum that might interfere with these pathways. Functional assays do not directly detect an AAb and only infer its activity through the effect observed on specific biological activity attributed to the cytokine. Thus, it is important to address the specificity of the assay by combining it with a ligand binding assay in order to demonstrate the presence of an AAb, and to utilize a control cytokine that uses the same signaling molecules in order to demonstrate neutralization of a specific cytokine. For instance, the assay for IFNγ AAbs makes use of IFNα as a control for specificity because both cytokines signal through STAT-1. In clinical practice, ligand binding assays may be used as a high throughput screen for anti-cytokine AAbs followed by a functional assay as confirmation.

As these assays make use of PBMCs, it is necessary to develop a pool of previously screened donors for the assay. Donor PBMCs for these assays are initially evaluated for their response to IFNγ and GM-CSF and the ability of previous characterized inhibitory serum to effectively neutralize the IFNγ or GM-CSF response to these cytokines. In our laboratory practice, freshly isolated PBMC from acceptable donors are utilized for clinical testing. It may also be possible to utilize cryopreserved PBMC from acceptable donors, however, we have not tested the performance of cryopreserved PBMC for phosphorylation assays extensively. An alternative strategy may be to utilize cell lines that are responsive to these cytokines.

## Diagnostic Utility

These functional assays enable the detection of functional, neutralizing AAbs to IFNγ or GM-CSF and have been validated for clinical use in a few clinical laboratories. As illustrated in Figure 1C, serum from a patient with extra-pulmonary, treatment-refractory NTM infection inhibited IFNγ-mediated phosphorylation of STAT1 while IFNα responses were unaffected (Figure 1D), indicating specificity for the IFNγ pathway. Using protein A purification for immunoglobulins, we demonstrated that the inhibitory component resides in the immunoglobulin fraction of patient's serum (63). In our experience, ELISA analysis of serum samples with known neutralizing activity demonstrated the presence of anti-IFNγ IgG AAbs, therefore proving that inhibition of the IFNγ pathway is IgG mediated. We have additionally shown that these autoantibodies are biologically significant because they abrogate the ability of *Listeria monocytogenes*-infected human monocytes to clear infection when activated by IFNγ (63). Similar to the flow cytometry assay for anti-IFNγ AAbs, inhibition of phosphorylation of STAT5 by serum from patients with clinically proven PAP following GM-CSF stimulation of PBMCs has been established as a clinical assay for functional evaluation of anti-GM-CSF AAbs. In our experience, as with anti-IFNγ AAbs, serum samples that had significant neutralizing activity for GM-CSF mediated phosphorylation of STAT5 also showed increased binding to GM-CSF in an ELISA, confirming that p-STAT5 inhibition by serum samples from these patients was antibody mediated.

## Clinical and Therapeutic Monitoring

These flow cytometry-based assays can not only be used to demonstrate the presence of anti-cytokine AAbs in serum samples, but can also be used to monitor the success of immune modulation. Encouraging evidence is emerging for the efficacy of rituximab, an anti-CD20- monoclonal antibody that leads to depletion of B lymphocytes, in IFNγ AAb-mediated acquired immunodeficiency (19, 63, 64). The efficacy of rituximab in inducing and maintaining disease remission has been described in several case reports of patients with anti-IFNγ autoantibodies and treatment-refractory NTM infection (19, 63, 65). Monitoring p-STAT1 expression in conjunction with B cell numbers and percentages is of utility in such patients in order to assess the efficacy of immune modulation. We and others have shown a correlation between the decrease in peripheral blood B cells and improvement in IFNγ-induced phosphorylation of STAT1 as an indication of the release of the IFNγ signaling pathway from anti-IFNγ AAb mediated inhibition (63).

Recombinant GM-CSF (subcutaneous or inhaled) and B cell depleting agents such as rituximab have been used to treat autoimmune PAP due to anti-GM-CSF autoantibodies (66–68). Monitoring anti-GM-CSF AAb levels and their neutralizing capability may be of utility along with the assessment of clinical parameters for determining the efficacy of treatment.

## Conclusions

Anti-cytokine AAbs causing immunodeficiency or dysregulation now form a distinct group of PIDs that share phenotypic characteristics with PIDs that occur due to pathogenic variants in genes encoding proteins involved in cytokine signaling pathways. As the management of patients with anti-cytokine AAbs differs from those with genetically disrupted cytokine signaling pathways, laboratory analysis for these AAbs is essential to enable not just improved phenotyping of these patients, but also to provide diagnostic and immune monitoring tools.

The neutralizing capability of anti-cytokine AAbs can be assessed by the analysis of the phosphorylation status of signaling molecules downstream of the relevant cytokine receptor. Such assays should be combined with ligand binding assays such as ELISA to demonstrate the presence of an antibody that correlates with neutralizing activity. Since signaling molecules such as STAT1 and STAT5 are phosphorylated by several cytokines, it is necessary to demonstrate specificity of these assays by utilizing alternate recombinant cytokine controls where possible. These assays that demonstrate the neutralizing capability of anti-cytokine AAbs are of utility in immune monitoring, particularly to demonstrate recovery of these cytokine pathways if immune depletion of B cells or plasmapheresis is employed to remove the source of the autoantibody or the autoantibody itself.

## Author Contributions

VK conceptualized and developed content for the manuscript. PM contributed to writing the manuscript. TL performed flow cytometry analysis of the AAb assays.

### Conflict of Interest Statement

The authors declare that the research was conducted in the absence of any commercial or financial relationships that could be construed as a potential conflict of interest.

## Abbreviations

AAbs: Autoantibodies
ELISA: Enzyme Linked Immunosorbent Assay
PID: Primary Immunodeficiency
PAP: Pulmonary Alveolar Proteinosis
NTM: Non-tuberculous Mycobacteria
IFNγ: Interferon gamma
GM-CSF: Granulocyte Macrophage Colony Stimulating Factor
STAT: Signal Transducer and Activator of Transcription.
